# The Mammalian Disaggregase Machinery: Hsp110 Synergizes with Hsp70 and Hsp40 to Catalyze Protein Disaggregation and Reactivation in a Cell-Free System

**DOI:** 10.1371/journal.pone.0026319

**Published:** 2011-10-14

**Authors:** James Shorter

**Affiliations:** Stellar-Chance Laboratories, Department of Biochemistry and Biophysics, University of Pennsylvania School of Medicine, Philadelphia, Pennsylvania, United States of America; Thomas Jefferson University, United States of America

## Abstract

Bacteria, fungi, protozoa, chromista and plants all harbor homologues of Hsp104, a AAA+ ATPase that collaborates with Hsp70 and Hsp40 to promote protein disaggregation and reactivation. Curiously, however, metazoa do not possess an Hsp104 homologue. Thus, whether animal cells renature large protein aggregates has long remained unclear. Here, it is established that mammalian cytosol prepared from different sources possesses a potent, ATP-dependent protein disaggregase and reactivation activity, which can be accelerated and stimulated by Hsp104. This activity did not require the AAA+ ATPase, p97. Rather, mammalian Hsp110 (Apg-2), Hsp70 (Hsc70 or Hsp70) and Hsp40 (Hdj1) were necessary and sufficient to slowly dissolve large disordered aggregates and recover natively folded protein. This slow disaggregase activity was conserved to yeast Hsp110 (Sse1), Hsp70 (Ssa1) and Hsp40 (Sis1 or Ydj1). Hsp110 must engage substrate, engage Hsp70, promote nucleotide exchange on Hsp70, and hydrolyze ATP to promote disaggregation of disordered aggregates. Similarly, Hsp70 must engage substrate and Hsp110, and hydrolyze ATP for protein disaggregation. Hsp40 must harbor a functional J domain to promote protein disaggregation, but the J domain alone is insufficient. Optimal disaggregase activity is achieved when the Hsp40 can stimulate the ATPase activity of Hsp110 and Hsp70. Finally, Hsp110, Hsp70 and Hsp40 fail to rapidly remodel amyloid forms of the yeast prion protein, Sup35, or the Parkinson's disease protein, alpha-synuclein. However, Hsp110, Hsp70 and Hsp40 enhanced the activity of Hsp104 against these amyloid substrates. Taken together, these findings suggest that Hsp110 fulfils a subset of Hsp104 activities in mammals. Moreover, they suggest that Hsp104 can collaborate with the mammalian disaggregase machinery to rapidly remodel amyloid conformers.

## Introduction

The successful functioning of all cells depends on proper protein folding [1]. Thus, cells maintain sophisticated protein homeostasis (proteostasis) networks to ensure that protein biogenesis is successful and that polypeptides effectively acquire, maintain and (if necessary) reacquire their functional native structure [2]. Molecular chaperones engage polypeptide chains to assist protein folding and prevent aggregation [3], degradation systems recognize and eliminate terminally misfolded proteins [4], and protein disaggregases reverse protein aggregation [5], [6]. Despite these safeguards, environmental stress, genetic background and aging can all synergize to overwhelm the proteostasis network and the consequences can be dire [7]. Indeed, protein misfolding and aggregation are intimately connected with a series of increasing prevalent and invariably fatal neurodegenerative disorders including Alzheimer's disease (AD), Parkinson's disease (PD) and Huntington's disease (HD) [1], [2], [3], [7], [8].

The paucity of treatment options for these disorders reflects, at least in part, a fundamental lack of basic understanding of the mechanisms that cells use to safely reverse protein aggregation. Indeed, protein disaggregases are perhaps the mostly poorly understood components of proteostasis networks. Bacteria and the vast majority of eukaryotes, including fungi, protozoa, chromista and plants [9], possess homologues of the AAA+ ATPase, Hsp104. Hsp104 collaborates with Hsp70 and Hsp40 to promote protein disaggregation and reactivation [5], [6], [10], [11]. This activity enables these organisms to respond rapidly to protein-misfolding stress (e.g. heat or chemical shock) by rescuing enzymatically active proteins from denatured, amorphous protein aggregates [12], [13], [14], [15], [16]. Thus, protein functionality is recovered rapidly without incurring the large energetic and temporal costs expended by degrading the protein and replacing it via transcription and translation.

Hsp104 from yeast can even remodel extremely stable, amyloid fibers [17], [18], [19], [20], [21], which adopt a highly ordered cross-beta conformation [1], [22]. This activity has enabled yeast to exploit prions (infectious amyloids) for advantageous purposes [23], [24], [25]. The amyloid-remodeling activity of Hsp104 does not appear to be strictly conserved, as the *E. coli* homologue ClpB does not appear to be able to remodel amyloid [18], [26], [27]. Curiously, however, Hsp104 does not have any metazoan homologues. Thus, whether or how animal cells might disaggregate and reactivate proteins has long remained unclear.

The loss of Hsp104 from metazoan lineages is abrupt. The choanoflagellate protist, *Monosiga brevicollis*, one of the most advanced pre-metazoans has a clear Hsp104 homologue [28], whereas even early branching metazoans like the sea anemone, *Nematostella vectensis* do not [29]. The reason underlying the loss of Hsp104 is unclear, especially because Hsp104 is well tolerated in animal systems. For example, transgenic mice expressing Hsp104 appear normal [30], [31] and Hsp104 increases stress tolerance of animal cells in culture [31], [32]. Moreover, Hsp104 rescues animal models of PD [17] and HD [30], [33], [34].

Whether animals even possess an analogous protein disaggregase, which functions to restore protein functionality remains unclear [6], [35]. Initial attempts to uncover such activities in mammalian cells have been unsuccessful [32]. More recently, crude *C. elegans* and mammalian extracts have been shown to possess a slow amyloid disaggregation activity that is tightly linked to protein degradation [36], [37], [38]. The identity of the disaggregase, however, which exhibits unusual properties such as resistance to high temperature (e.g. 80°C), remains unclear [36], [37], [38].

The search for functional equivalents of Hsp104 has led researchers to consider other members of the diverse AAA+ protein family. It has been suggested that one highly conserved candidate, p97 (also known as VCP or Cdc48), might perform a disaggregase function [39]. Indeed, p97 can act as a molecular chaperone and prevent protein aggregation [40], [41], and even appears to regulate some amyloid misfolding events in a manner similar to Hsp104 [42]. Moreover, p97 can collaborate with Hsp70 and Hsp40 to refold soluble misfolded conformers [43]. However, no convincing demonstration of p97-catalyzed disaggregation has been forthcoming using pure components.

It has also been suggested that in the absence of Hsp104, high concentrations of metazoan Hsp70 and Hsp40 might suffice to promote protein disaggregation [44], [45], [46]. However, Hsp70 and Hsp40 are unable to rapidly remodel amyloid [17], [20], [47] and fail to disaggregate large amorphous protein aggregates [10], [32], [44], [46]. Thus, it continues to remain unclear whether protein disaggregation and reactivation are ever coupled in metazoa, or whether metazoan proteostasis is more centered on clearing aggregated proteins via autophagy and other proteolytic systems [4].

Here, a potent metazoan disaggregase and renaturation machinery is uncovered using a cell-free system. It is established that mammalian cytosol can disaggregate and reactivate aggregated proteins in an ATP-dependent manner. Hsp70 and Hsp40 chaperones play key roles in this process. However, using pure components it is demonstrated that Hsp70 and Hsp40 are not sufficient. Indeed, it is established that an Hsp70-related chaperone with Hsp70 nucleotide exchange activity, Hsp110 [48], [49], [50], [51], [52], [53], [54], [55], [56], is also needed. Hsp110 synergizes with Hsp70 and Hsp40 to drive protein disaggregation and reactivation in mammalian cytosol and in a minimal pure protein setting.

## Results and Discussion

### Mammalian cytosol possesses ATP-dependent disaggregase activity that is enhanced by Hsp104

**Figure 1:**
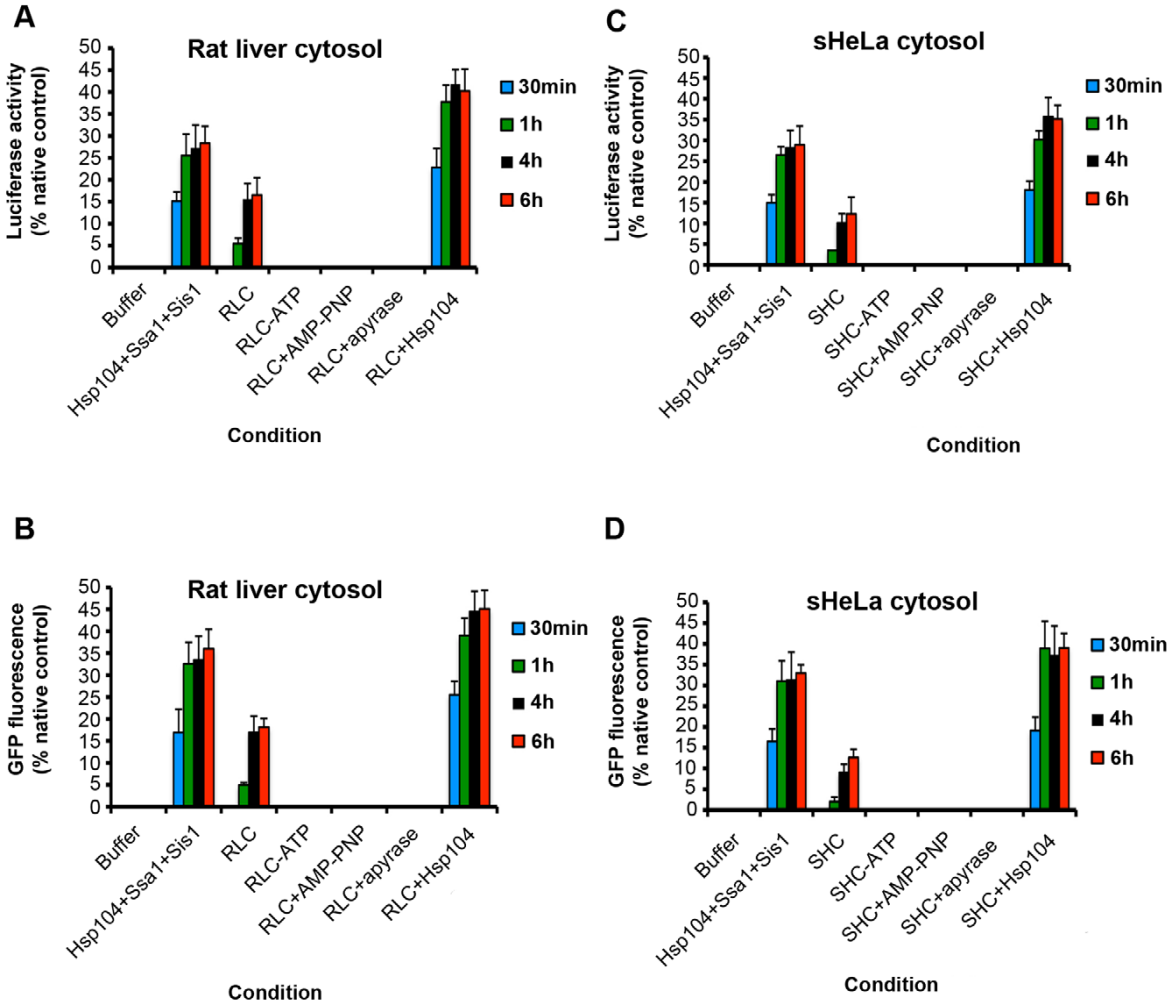
Mammalian cytosol contains an ATPase-dependent disaggregation machinery. (**A, B**) Urea-denatured luciferase aggregates (50 nM) (**A, C**) or heat-denatured GFP aggregates (0.45 µM) (**B, D**) were incubated for 30 min (blue bars), 1 h (green bars), 4 h (black bars), or 6 h (red bars) at 25°C in buffer plus ATP (5 mM) and ATP-regeneration system without or with the indicated combination of Hsp104 (1 µM), Ssa1 (1 µM), Sis1 (1 µM), RLC (10 mg/ml) (**A, B**), or SHC (10 mg/ml) (**C, D**). In the indicated reactions, ATP was omitted or replaced with AMP-PNP. Alternatively, ATP was included but the ATP-regeneration system was replaced with apyrase. Disaggregation and reactivation of luciferase was monitored by luminescence. Luminescence measurements were converted into reactivation yield (% of the maximum recoverable luciferase activity) by comparison to the luminescence of known quantities of soluble, native luciferase (**A, C**). Disaggregation and reactivation of GFP was monitored by fluorescence. Fluorescence measurements were converted into reactivation yield (% of the maximum recoverable GFP fluorescence) by comparison to the fluorescence of known quantities of soluble, native GFP (**B, D**). Values represent means ± SEM (n = 3).

First, it was tested whether cytosol preparations from two mammalian sources, rat liver and sHeLa cells, possessed any intrinsic protein disaggregation and reactivation activity. Two classic model aggregated substrates that are widely employed for protein disaggregation experiments with Hsp104 and ClpB were used: urea-denatured luciferase, which forms aggregated structures of ∼500–2,000 kDa and greater in size [10], and heat-denatured GFP, which forms a continuum of aggregated structures of ∼500 kDa and greater in size [57], [58], [59]. For comparison, purified Hsp104, Ssa1 and Sis1 from yeast were employed, which rapidly disaggregated both luciferase (Figure 1A, C) and GFP (Figure 1B, D) within 30 min to 1 h. By contrast, neither rat liver cytosol (RLC) nor sHeLa cytosol (SHC) could disaggregate and reactivate significant amounts of luciferase (Figure 1A, C) or GFP (Figure 1B, D) in this time frame. Addition of Hsp104 to RLC or SHC stimulated rapid disaggregation and reactivation of luciferase (Figure 1A, C) and GFP (Figure 1B, D), indicating that Hsp104 is fully competent to collaborate with the mammalian proteostasis machinery.

Importantly, RLC and SHC were not completely inactive. After longer incubations of 4 h or more, both RLC and SHC reactivated luciferase (Figure 1A, C) and GFP (Figure 1B, D). RLC (Figure 1A, B) possessed slightly greater activity than SHC (Figure 1C, D). This activity depended upon ATP hydrolysis, and was not observed when cytosol was not supplemented with ATP or if AMP-PNP (a non-hydrolyzable ATP analogue) was supplemented in place of ATP, or if ATP was depleted by apyrase treatment (Figure 1A-D). Thus, the mammalian cytosol harbors an ATPase-dependent protein disaggregation activity.

### p97 is not required for the disaggregase activity

**Figure 2:**
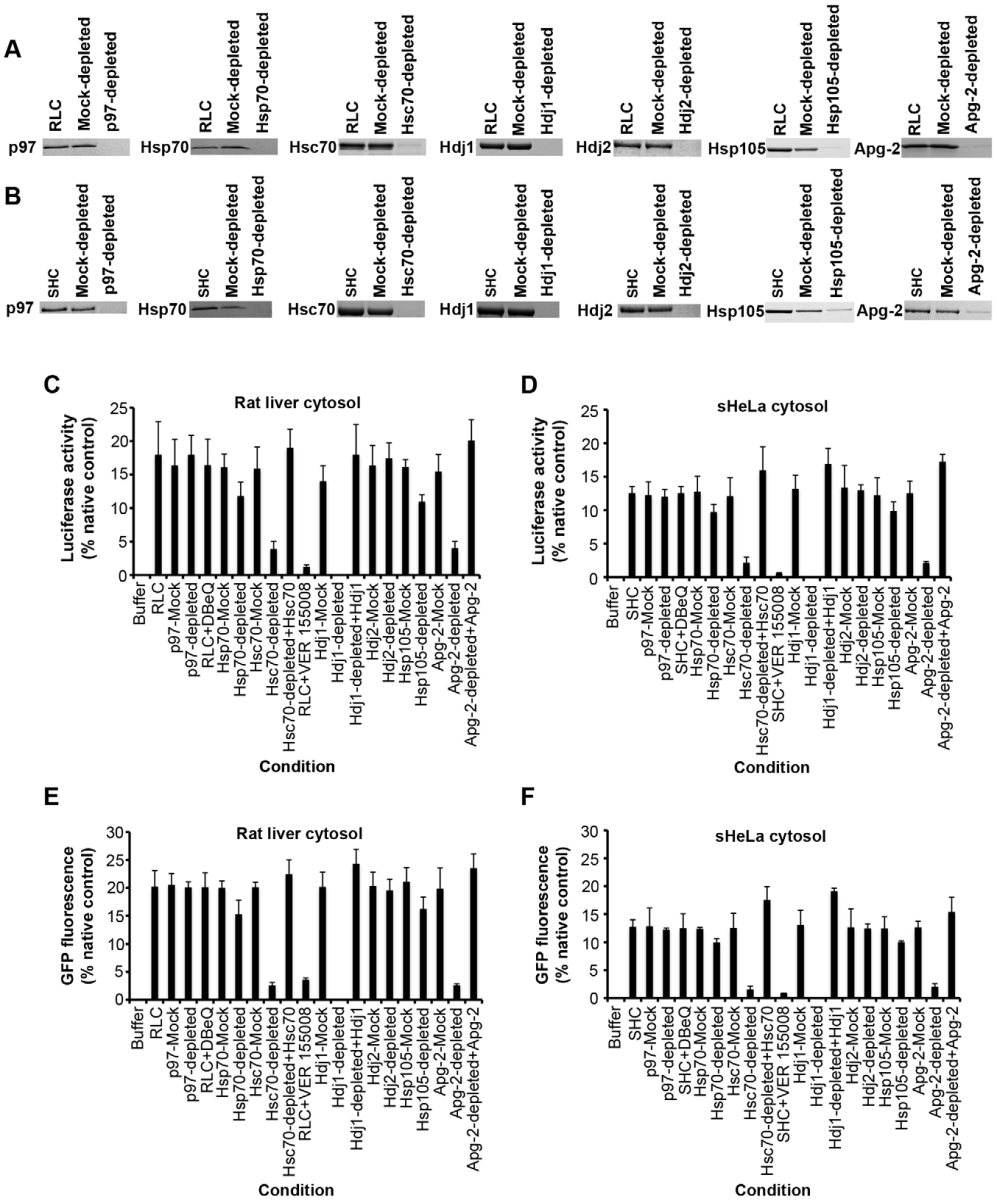
Hsp110, Hsp70 and Hsp40 are key components of the mammalian disaggregation machinery. (**A, B**) Immunoblots showing depletion of either p97, Hsp70, Hsc70, Hdj1, Hdj2, Hsp105 or Apg-2 from either RLC (**A**) or SHC (**B**) as described in Materials and Methods. 20 µg of cytosol was fractionated by SDS-PAGE and processed for immunoblot. (**C–F**) Urea-denatured luciferase aggregates (50 nM) (**C, D**) or heat-denatured GFP aggregates (0.45 µM) (**E, F**) were incubated for 6 h in buffer plus ATP (5 mM) and ATP-regeneration system without or with the indicated cytosol (10 mg/ml), mock-depleted cytosol (10 mg/ml) or depleted cytosol (10 mg/ml). In the indicated reactions, the small molecule p97 inhibitor, DBeQ (100 µM), or Hsp70 inhibitor, VER 155008 (100 µM), were included. In the indicated reactions, Hsc70-depleted cytosol was supplemented with Hsc70, Hdj1-depleted cytosol was supplemented with Hdj1 and Apg-2-depleted cytosol was supplemented with Apg-2 as described in Materials and Methods. Disaggregation and reactivation of luciferase was monitored by luminescence. Luminescence measurements were converted into reactivation yield (% of the maximum recoverable luciferase activity) by comparison to the luminescence of known quantities of soluble, native luciferase (**C, D**). Disaggregation and reactivation of GFP was monitored by fluorescence. Fluorescence measurements were converted into reactivation yield (% of the maximum recoverable GFP fluorescence) by comparison to the fluorescence of known quantities of soluble, native GFP (**E, F**). Values represent means ± SEM (n = 3).

A candidate-based approach was employed to identify the ATP-dependent disaggregases. First, the AAA+ ATPase, p97, was considered. p97 was depleted by greater than 98% from RLC and SHC, using a p97-binding fragment of Ufd1, UT6, fused to GST (Figure 2A, B) [60]. Both p97-depleted RLC and p97-depleted SHC could disaggregate and reactivate luciferase (Figure 2C, D) and GFP (Figure 2E, F). A small molecule, DBeQ, which potently and selectively inhibits the ATPase activity of p97 [61], had no effect on protein disaggregation by either RLC (Figure 2C, E) or SHC (Figure 2D, F). Thus, p97 is unlikely to contribute to the observed disaggregase activity.

### The Hsp70 chaperone system is a key component of the disaggregase machinery

Next, the Hsp70 chaperone system was considered. First, Hsp70 (a heat-inducible Hsp70 that is not expressed at high levels under non-stressful conditions) was immunodepleted by greater than 98% (Figure 2A, B). Hsp70 immunodepletion had only a slight effect on the disaggregase activity of RLC and SHC (Figure 2C–F). By contrast, immunodepletion of Hsc70 (the constitutive Hsp70 chaperone) by greater than 95% (Figure 2A, B), severely reduced the disaggregase activity of RLC and SHC (Figure 2C–F). Importantly, adding pure Hsc70 back to Hsc70-depleted cytosol restored full disaggregase activity (Figure 2C–F). Thus, Hsc70 itself drives the disaggregase activity, and not other factors that may have been co-depleted during immunodepletion of Hsc70. Moreover, a small molecule inhibitor of Hsp70 and Hsc70 ATPase activity, VER 155008 [62], inhibited the disaggregation of activity of RLC and SHC (Figure 2C–F). These data suggest that the Hsp70 chaperone system is a critical component of the mammalian disaggregation machinery.

Next, the role of Hsp40 co-chaperones was assessed. Hdj1 or Hdj2 were immunodepleted from RLC (Figure 2A) and SHC (Figure 2B) by ∼100%. Depletion of Hdj2 had no effect on disaggregase activity (Figure 2C–F), whereas depletion of Hdj1 eliminated activity (Figure 2C–F). Importantly, adding pure Hdj1 back to Hdj1-depleted cytosol restored full disaggregase activity, reinforcing the importance of Hdj1 itself (Figure 2C–F). These data suggest that Hsc70 and Hdj1 are key components of the mammalian disaggregase machinery.

### Mammalian Hsp70 and Hsp40 are not sufficient for robust protein disaggregation

**Figure 3:**
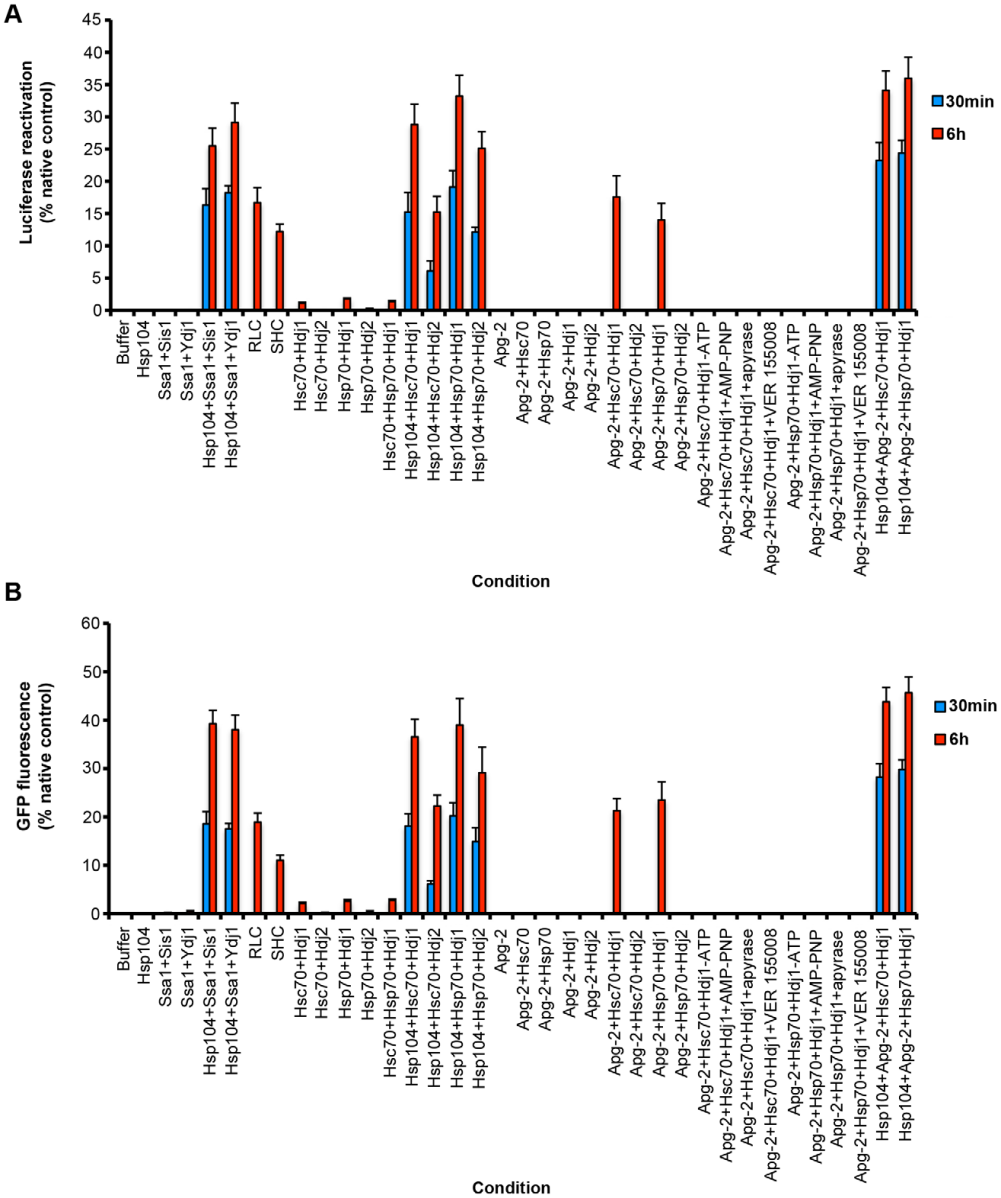
Pure Hsp110, Hsp70 and Hsp40 drive protein disaggregation. (**A, B**) Urea-denatured luciferase aggregates (50 nM) (**A**) or heat-denatured GFP aggregates (0.45 µM) (**B**) were incubated for 30 min (blue bars) or 6 h (red bars) at 37°C in buffer plus ATP (5 mM) and ATP-regeneration system without or with the indicated combination of Hsp104 (1 µM), Ssa1 (1 µM), Ydj1 (1 µM), RLC (10 mg/ml), SHC (10 mg/ml), Hsc70 (1 µM), Hdj1 (1 µM), Hdj2 (1 µM), Hsp70 (1 µM) and Apg-2 (1 µM). In the indicated reactions, ATP was omitted or replaced with AMP-PNP. Alternatively, ATP was included but the ATP-regeneration system was replaced with apyrase. The small molecule inhibitor VER 155008 (100 µM) was included in the indicated reactions. Disaggregation and reactivation of luciferase was monitored by luminescence. Luminescence measurements were converted into reactivation yield (% of the maximum recoverable luciferase activity) by comparison to the luminescence of known quantities of soluble, native luciferase (**A**). Disaggregation and reactivation of GFP was monitored by fluorescence. Fluorescence measurements were converted into reactivation yield (% of the maximum recoverable GFP fluorescence) by comparison to the fluorescence of known quantities of soluble, native GFP (**B**). Values represent means ± SEM (n = 3).

The previous experiments defined Hsc70 and Hdj1 as key components of the disaggregase machinery present in mammalian cytosol. Could this machinery be reconstituted using pure proteins? Thus, the ability of Hsc70∶Hdj1, Hsc70∶Hdj2, Hsp70∶Hdj1 and Hsp70∶Hdj2 pairs to disaggregate luciferase and GFP was tested. Consistent, with the immunodepletion studies (Figure 2), Hsc70∶Hdj2 and Hsp70∶Hdj2 did not disaggregate luciferase (Figure 3A) or GFP (Figure 3B). By contrast, Hsc70∶Hdj1 and Hsp70∶Hdj1 displayed some disaggregase activity (Figure 3A, B). However, the recovery of folded luciferase and GFP by Hsc70∶Hdj1 or Hsp70∶Hdj1 was ∼10-fold less than that achieved by RLC or SHC (Figure 3A, B), or by Hsp104, Ssa1 and Sis1 (Figure 3A, B). Mixing Hsc70∶Hdj1 and Hsp70∶Hdj1 did not increase activity any further (Figure 3A, B). Moreover, increasing the Hsc70∶Hdj1 and Hsp70∶Hdj1 concentration 20-fold also failed to increase disaggregase activity. These data suggested that additional factors were needed to reconstitute the full disaggregase activity of RLC and SHC.

### Hsp104 synergizes with mammalian Hsp70∶Hsp40 pairs to promote protein disaggregation

Interestingly, Hsc70∶Hdj1 and Hsp70∶Hdj1 were more active in protein disaggregation than the yeast Hsp70∶Hsp40 pairs: Ssa1∶Ydj1 and Ssa1∶Sis1 (Figure 3A, B). These data indicate that the mammalian Hsp70 chaperone system might have evolved greater protein disaggregase activity in the absence of an Hsp104 homologue. Hsp104 could synergize with all the mammalian Hsp70∶Hsp40 pairs tested here: Hsc70∶Hdj1, Hsc70∶Hdj2, Hsp70∶Hdj1 and Hsp70∶Hdj2 to promote rapid luciferase and GFP disaggregation and reactivation (Figure 3A, B). These data suggested that Hsp104 could collaborate with various mammalian Hsp70 and Hsp40 chaperones. Moreover, the disaggregase activity of Hsp104 with Hsc70∶Hdj1 or Hsp70∶Hdj1 was very similar to that of Hsp104 with Ssa1∶Sis1 (Figure 3A, B). Thus, in contrast to the inability of Hsp104 to collaborate with prokaryotic Hsp70 (DnaK) [10] there does not appear to be a ‘species barrier’ between Hsp104 and mammalian chaperones, which helps explain why Hsp104 can confer therapeutic benefits for protein misfolding disorders in several metazoan settings [17], [30], [32], [33], [34].

### Hsp110 is an essential component of the cytosolic disaggregase machinery

The ability of Hsp104 to synergize with Hsc70∶Hdj1 and Hsp70∶Hdj1, and the low activity of these Hsp70∶Hsp40 pairs compared to RLC or SHC, suggested that other factors in mammalian cytosol might contribute to protein disaggregation. One obvious candidate was Hsp110, a divergent relative of Hsp70 that can stabilize unfolded proteins [63], [64], [65] and serve as a nucleotide exchange factor for Hsp70 [54], [56]. Moreover, Hsp110 may co-operate with Hsp70 to assist protein folding [53]. Thus, two Hsp110 variants: Hsp105, a heat-inducible Hsp110 [66], or Apg-2, a constitutive Hsp110 [67], were immunodepleted by greater than ∼93% from RLC (Figure 2A) and SHC (Figure 2B). Hsp105-depleted cytosol had slightly reduced luciferase and GFP disaggregase activity, whereas Apg-2-depleted cytosol had greatly reduced disaggregase activity (Figure 2C–F). In fact, Apg-2 depletion reduced disaggregase activity (Figure 2C–F) to levels provided by Hsc70∶Hdj1 or Hsp70∶Hdj1 in isolation (Figure 3A, B). Addition of pure Apg-2 back to Apg-2-depleted RLC or SHC restored disaggregase activity to that of untreated or mock-depleted cytosol (Figure 2C–F), indicating that Apg-2 itself was a critical determinant supporting protein disaggregation.

### Pure Hsp110 synergizes the Hsp70 and Hsp40 to promote protein disaggregation in a minimal system

Next, Apg-2 was added to pure protein disaggregation assays. Apg-2 synergized with Hsc70∶Hdj1 and Hsp70∶Hdj1 to promote disaggregation of luciferase (Figure 3A) and GFP (Figure 3B). The addition of Apg-2 increased disaggregase activity to levels approaching that of RLC and SHC (Figure 3A, B). Notably, Apg-2 did not catalyze protein disaggregation alone or in combination with Hsc70, Hsp70 or Hdj1 alone (Figure 3A, B). Furthermore, Apg-2 did not synergize with Hsc70∶Hdj2 or Hsp70∶Hdj2 (Figure 3A, B). Importantly, just like the disaggregase activity of RLC and SHC, the disaggregase activity of Apg-2:Hsc70∶Hdj1 or Apg-2:Hsc70∶Hdj1 was inhibited by the absence of adenine nucleotide, by AMP-PNP, when ATP was depleted with apyrase, and by the small molecule Hsp70 inhibitor VER 155008 (Figure 3A, B). Apg-2 did not stimulate disaggregase activity as much or as rapidly as Hsp104 (Figure 3A, B). However, the combination of Hsp104, Apg-2 with either Hsc70∶Hdj1 or Hsp70∶Hdj1, yielded the most efficacious protein disaggregation (Figure 3A, B). Together these data clearly establish that, even in the absence of Hsp104, mammalian Hsp110 synergizes with Hsp70 and Hsp40 to promote the disaggregation and reactivation of large protein aggregates.

### Hsp110 disaggregase activity is conserved to yeast Sse1

**Figure 4:**
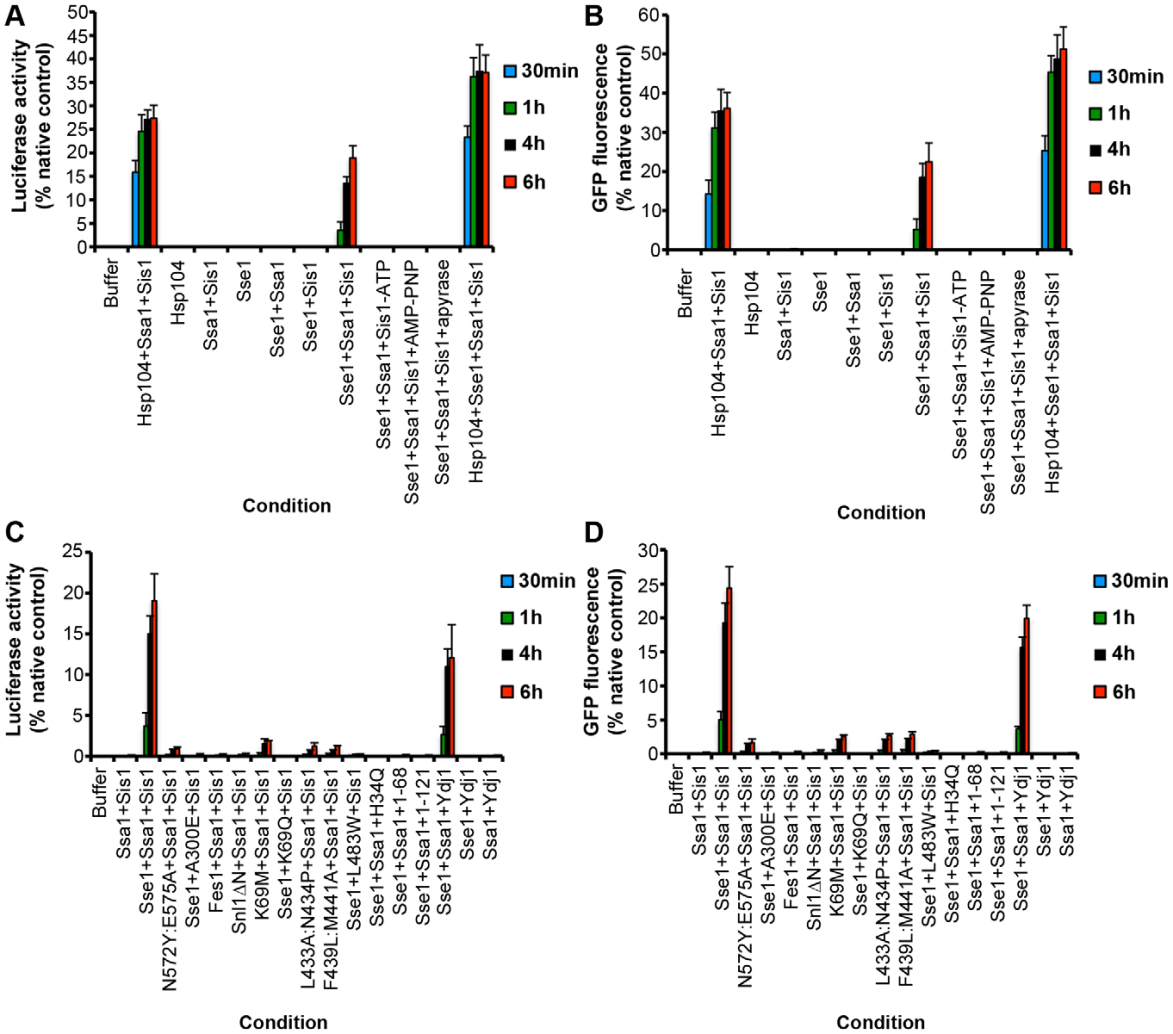
Hsp110 NEF activity, ATPase activity and substrate-binding activity are essential for protein disaggregation. (**A, B**) Urea-denatured luciferase aggregates (50 nM) (A) or heat-denatured GFP aggregates (0.45 µM) (B) were incubated for 30 min (blue bars), 1 h (green bars), 4 h (black bars), or 6 h (red bars) at 25°C in buffer plus ATP (5 mM) and ATP-regeneration system without or with the indicated combination of Hsp104 (1 µM), Ssa1 (1 µM), Sis1 (1 µM) and Sse1 (1 µM). In the indicated reactions, ATP was either omitted or replaced with AMP-PNP. Alternatively, ATP was included but the ATP-regeneration system was replaced with apyrase. Disaggregation and reactivation of luciferase was monitored by luminescence. Luminescence measurements were converted into reactivation yield (% of the maximum recoverable luciferase activity) by comparison to the luminescence of known quantities of soluble, native luciferase (**A**). Disaggregation and reactivation of GFP was monitored by fluorescence. Fluorescence measurements were converted into reactivation yield (% of the maximum recoverable GFP fluorescence) by comparison to the fluorescence of known quantities of soluble, native GFP (B). Values represent means ± SEM (n = 3). (**C, D**) Urea-denatured luciferase aggregates (50 nM) (**C**) or heat-denatured GFP aggregates (0.45 µM) (**D**) were incubated for 30 min (blue bars), 1 h (green bars), 4 h (black bars), or 6 h (red bars) at 25°C in buffer plus ATP (5 mM) and ATP-regeneration system without or with the indicated combination of Ssa1 (1 µM), Sis1 (1 µM), Sse1 (1 µM), Sse1^N572Y:E575A^ (1 µM, a NEF-defective Sse1 mutant), Ssa1^A300E^ (1 µM, an Ssa1 mutant unable to interact with Sse1), Fes1 (1 µM, a Ssa1 NEF), Snl1ΔN (1 µM, a Ssa1 NEF), Sse1^K69M^ (1 µM, an ATPase-dead Sse1 mutant), Ssa1^K69Q^ (1 µM, an ATPase-dead Ssa1 mutant), Sse1^L433A:N434P^ (1 µM, a substrate-binding defective Sse1 mutant), Sse1^F439L:M441A^ (1 µM, a substrate-binding defective Sse1 mutant), Ssa1^L483W^ (1 µM, a substrate-binding defective Ssa1 mutant), Sis1^H34Q^ (1 µM, a Sis1 mutant unable to stimulate Ssa1 ATPase activity), Sis1^1–68^ (1 µM, the J domain of Sis1), Sis1^1–121^ (1 µM, the J and G/F-domains of Sis1) and Ydj1 (1 µM, an Hsp40). Disaggregation and reactivation of luciferase was monitored by luminescence. Luminescence measurements were converted into reactivation yield (% of the maximum recoverable luciferase activity) by comparison to the luminescence of known quantities of soluble, native luciferase (C). Disaggregation and reactivation of GFP was monitored by fluorescence. Fluorescence measurements were converted into reactivation yield (% of the maximum recoverable GFP fluorescence) by comparison to the fluorescence of known quantities of soluble, native GFP (D). Values represent means ± SEM (n = 3).

The ability of Hsp110 to synergize with Hsp70 and Hsp40 to promote protein disaggregation and reactivation was conserved to the yeast Hsp110 homologue, Sse1. Thus, Sse1 synergized with Ssa1∶Sis1 to promote disaggregation of luciferase (Figure 4A) and GFP (Figure 4B). Here too, disaggregation proceeded with slower kinetics than in the presence of Hsp104, Ssa1 and Sis1 and lower amounts of folded luciferase and GFP could be recovered (Figure 4A, B). Sse1 was inactive alone or when paired with Ssa1 alone or Sis1 alone (Figure 4A, B). Moreover, the activity of Sse1∶Ssa1∶Sis1 was inhibited by the absence of adenine nucleotide, by AMP-PNP, or when ATP was depleted with apyrase (Figure 4A, B). Interestingly, addition of Sse1 to Hsp104, Ssa1 and Sis1 yielded a more rapid and potent disaggregase activity (Figure 4A, B).

### Sse1 must bind Ssa1, substrate and hydrolyze ATP to promote protein disaggregation

The ability of Sse1 to synergize with Ssa1∶Sis1 to promote protein disaggregation allowed an assessment of the importance of Sse1 NEF activity, ATPase activity and substrate-binding activity for disaggregation by employing well-defined Sse1 mutants [53]. First, it was tested whether the NEF activity of Sse1 was important for protein disaggregation. A Sse1 mutant without NEF activity, Sse1^N572Y,E575A^ [53], failed to promote luciferase and GFP disaggregation by Ssa1∶Sis1 (Figure 4C, D). Similarly, an Ssa1 mutant, Ssa1^A300E^ [53], which cannot engage Sse1, could not promote protein disaggregation (Figure 4C, D). Thus, Sse1 and Ssa1 must interact in a manner that enables Ssa1 nucleotide exchange to promote protein disaggregation. However, NEF activity alone was not sufficient. Two other Ssa1 NEFs: Fes1 [68] and Snl1ΔN [69], [70] could not substitute for Sse1 in the promotion of protein disaggregation (Figure 4C, D).

These data suggested that Sse1 was not functioning as a simple NEF to promote protein disaggregation. To assess other Sse1 functionalities, we employed Sse1^K69M^, which has defective ATPase activity [49]. Sse1^K69M^, Ssa1 and Sis1 failed to promote luciferase (Figure 4C) or GFP disaggregation (Figure 4D). Sse1^K69M^ is fully functional as a Ssa1 NEF [56]. Thus, these data indicate that Sse1 must do more than simply promote Ssa1 nucleotide exchange to promote disaggregation. An ATPase defective Ssa1 variant, Ssa1^K69Q^ [71], also eliminated disaggregase activity (Figure 4C, D). Thus, both Ssa1 and Sse1 must hydrolyze ATP to promote protein disaggregation.

Next, Sse1 variants bearing mutations in the putative peptide-binding site were assessed [53]. Sse1^L443A, N434P^ and Sse1^F439L, M441A^ both failed to promote luciferase or GFP disaggregation by Ssa1 and Sis1 (Figure 4C, D). Similarly, an Ssa1 variant defective in substrate binding, Ssa1^L483W^ [72], eliminated disaggregase activity (Figure 4C, D). Thus, both Sse1 and Ssa1 must engage substrate to promote protein disaggregation.

### Defining the role of Hsp40 in the disaggregation reaction

Next, the role of Sis1 in the disaggregation reaction was probed. Must Sis1 stimulate Hsp70 ATPase activity to promote disaggregation? To answer this question, a J domain mutant, Sis1^H34Q^, which is unable to stimulate Hsp70 ATPase activity was employed [73]. Sis1^H34Q^ failed to support protein disaggregation (Figure 4C, D), indicating that the ability of Sis1 to stimulate Hsp70 ATPase activity is important for protein disaggregation. However, a functional J domain was not sufficient: Sis1 truncation mutants bearing only the J domain, Sis1^1–68^ [73], or the J domain plus the G/F domain, Sis1^1–121^ [73], could not support protein disaggregation (Figure 4C, D). Thus, Sis1 must perform other functions beyond stimulating Hsp70 ATPase activity to promote protein disaggregation.

Sis1 can stimulate Ssa1 and Sse1 ATPase activity, whereas another yeast Hsp40, Ydj1, stimulates Ssa1 ATPase activity but not Sse1 ATPase activity [49]. To test the importance of the ability of Sis1 to stimulate Sse1 ATPase activity for protein disaggregation, Sis1 was substituted with Ydj1. Sse1, Ssa1 and Ydj1 supported protein disaggregation, but not as effectively as Sse1, Ssa1 and Sis1 (Figure 4C, D). Thus, one key role of the Hsp40 appears to be to stimulate Ssa1 ATPase activity, whereas the stimulation of Sse1 ATPase activity appears to be less important.

### Hsp110, Hsp70 and Hsp40 do not rapidly remodel amyloid

**Figure 5:**
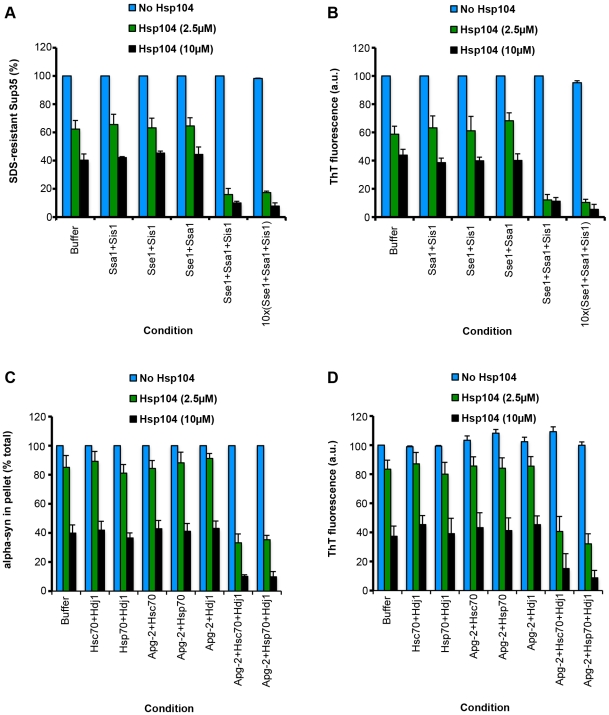
Pure Hsp110, Hsp70 and Hsp40 enhance Hsp104 disaggregase activity against Sup35 prions and α-syn amyloid fibers. (**A, B**) Preformed Sup35 prions (2 µM monomer) were incubated for 6 h at 25°C in buffer plus ATP (5 mM) and ATP-regeneration system without or with the indicated combination of Ssa1 (2 µM), Sis1 (2 µM), Sse1 (2 µM) (each individual combination is an individual category of the x-axis) in the absence (blue bars) or presence of Hsp104 (0.5 µM, green bars; or 2 µM, black bars). For the 10x(Sse1+Ssa1+Sis1) condition the concentration of Ssa1, Sis1 and Sse1 was increased to 20 µM. Sup35 prion disassembly was assessed by the amount of SDS-resistant Sup35 (**A**) or by ThT fluorescence (**B**). Values represent means ± SEM (n = 3). (**C, D**) Preformed amyloid fibers composed of α-syn (0.5 µM monomer) were incubated for 6 h at 37°C in buffer plus ATP (5 mM) and ATP-regeneration system without or with the indicated combination of Hsc70 (10 µM), Hdj1 (10 µM), Hsp70 (10 µM) and Apg-2 (10 µM) (each individual combination is an individual category of the x-axis) in the absence (blue bars) or presence of Hsp104 (2.5 µM, green bars; or 10 µM, black bars). Fiber integrity was then determined by sedimentation analysis (**C**) or by ThT fluorescence (**D**). Values represent means ± SEM (n = 3).

Could Hsp110, Hsp70 and Hsp40 disaggregate amyloid conformers? Sse1, Ssa1 and Sis1 were tested against infectious amyloid forms of the yeast prion protein, Sup35 [19]. SDS-resistance and Thioflavin-T (ThT) fluorescence were employed to monitor Sup35 prion integrity [19], [20], [74]. Sse1, Ssa1 and Sis1 were ineffective in remodeling Sup35 prions (Figure 5A, B). Even a tenfold excess of Sse1, Ssa1, and Sis1 over Sup35 had no effect (Figure 5A, B). Moreover, increasing the reaction time to 24 h did not yield any disassembly. By contrast, Hsp104 could remodel Sup35 prions in the absence of Sse1, Ssa1 and Sis1 (Figure 5A, B) [19], [20]. Addition of binary combinations of Sse1, Ssa1 and Sis1 had little effect on Hsp104 activity (Figure 5A, B). By contrast, addition of Sse1, Ssa1 and Sis1 yielded greater Sup35 prion remodeling by Hsp104 (Figure 5A, B). These data are consistent with in vivo observations that Sse1 promotes Sup35 prion fragmentation by Hsp104 [75], [76]. Thus, although ineffective alone, Sse1, Ssa1 and Sis1 can enhance Sup35 prion remodeling by Hsp104.

Next, it was tested whether Apg-2:Hsc70∶Hdj1 or Apg-2:Hsp70∶Hdj1 could remodel amyloid conformers comprised of the PD protein α-synuclein (α-syn). Here too, Apg-2:Hsc70∶Hdj1 or Apg-2:Hsp70∶Hdj1 were ineffective alone (Figure 5C, D). By contrast, Hsp104 rapidly remodeled α-syn fibers in a manner that was enhanced by the addition of Apg-2:Hsc70∶Hdj1 or Apg-2:Hsp70∶Hdj1 (Figure 5C, D). Taken together these data suggest that although capable of slowly resolving large denatured protein aggregates, Hsp110, Hsp70 and Hsp40 fail to rapidly remodel amyloid. Thus, it appears as though they only fulfill a subset of Hsp104 disaggregase activities. However, Hsp104 can synergize with mammalian Hsp110, Hsp70 and Hsp40 to promote the disaggregation of amyloid conformers (Figure 5C, D) and amorphous, disordered aggregates (Figure 3A, B).

### Hsp110, Hsp70 and Hsp40: a mammalian disaggregase system

Collectively, these findings suggest that Hsp110, Hsp70 and Hsp40 constitute a disaggregase machinery active in the mammalian cytosol, which is able to recover natively folded proteins from large chemically or thermally denatured protein aggregates. This activity appears to be specific for disordered, amorphous aggregates. Hsp110, Hsp70 and Hsp40 were unable to disaggregate amyloid conformers. Thus, the metazoan amyloid disaggregation activity uncovered by Cohen, Dillin, Kelly and colleagues, which is tightly coupled to protein degradation rather than protein reactivation, is likely to be composed of different components [36], [37], [38]. Indeed, it may be that metazoa rely primarily on autophagy or other lysosomal processes to clear amyloid conformers that accumulate in the cytoplasm [4]. Nonetheless, it is now clear that metazoa have disaggregase systems in place that can couple protein disaggregation to protein renaturation or protein degradation. Activating these pathways might prove to be a useful therapeutic strategy against numerous disorders connected with protein misfolding and aggregation [1], [2], [8].

The disaggregase activity of Hsp110, Hsp70 and Hsp40 was conserved to the yeast homologues. Thus, Sse1, Ssa1 and Sis1 could synergize to rescue proteins from large disordered aggregates. This activity only allowed a slow rescue of active protein, in contrast to Hsp104-catalyzed reactions. These slow kinetics might explain why Sse1, Ssa1 and Sis1 cannot provide thermotolerance to yeast that lack Hsp104. Rather, the rapid protein disaggregation and reactivation catalyzed by Hsp104 appears critical for stress tolerance in vivo [12]. Notably, however, Hsp104 was more effective when combined with Sse1, Ssa1 and Sis1 than with Ssa1 and Sis1 alone. Thus, Sse1 assists Hsp104-catalyzed disaggregation.

Using a series of well-defined Sse1 mutants [53], it was evident that Sse1 must engage substrate, engage Hsp70, promote nucleotide exchange on Hsp70, and hydrolyze ATP to promote disaggregation of disordered aggregates. The requirement for all of these Sse1 functionalities distinguishes Sse1's function in protein disaggregation from its role in preventing protein aggregation, where the ATPase activity is dispensable [63]. Similarly, the role of Sse1 in assisting Ssa1 and Ydj1 in refolding soluble misfolded luciferase does not require Sse1 ATPase activity and the requirement for Sse1-peptide interactions is less stringent [53]. Here, the Hsp70 NEF activity is paramount [53]. For protein disaggregation, the Sse1 NEF activity is also essential, but it is not sufficient as neither Fes1 nor Snl1ΔN (two Ssa1 NEFs) could substitute for Sse1. Ssa1 must also hydrolyze ATP, engage substrate and Sse1 to promote protein disaggregation. Furthermore, protein disaggregation was most effective when the Hsp40 stimulated both Ssa1 and Sse1 ATPase activity. However, a J domain was not sufficient indicating that Hsp40 must perform other functions beyond stimulating Hsp70 and Hsp110 ATPase activity. Taken together, these data suggest that the synergy between Sse1, Ssa1 and Sis1 in protein disaggregation might reflect a coupling of Sse1 and Ssa1 ATPase activity to co-operative substrate handling, which gradually dissociates proteins from the aggregate surface. Thus, Hsp110 might potentiate ‘entropic pulling’ by Hsp70 [45], [77].

Although Hsp110, Hsp70 and Hsp40 were unable to remodel amyloid conformers, they did permit more effective amyloid remodeling by Hsp104. For example, Hsp104 more effectively eradicated amyloid forms of α-syn in the presence of Apg-2, Hsc70 and Hdj1 than in their absence. The ability of Hsp104 to interface with the mammalian disaggregase machinery and enable effective clearance of amyloid conformers might hold promise for applying Hsp104 to various devastating protein-misfolding disorders [6],[35].

## Materials and Methods

### Cytosol preparation

Rat liver cytosol (RLC) [78] and sHeLa interphase cytosol [79], [80] were prepared as described, except that protease inhibitors (complete cocktail (Roche) and 5 µM pepstatin A (Sigma)) were included in all buffers and at the final step cytosol was buffer exchanged into either luciferase refolding buffer (LRB: 25 mM HEPES-KOH pH 7.4, 150 mM KAOc, 10 mM MgAOc, 10 mM DTT) plus ATP (5 mM) or GFP refolding buffer (GRB: 25 mM HEPES-KOH, pH 7.4, 100 mM KCl, 20 mM MgCl_2_, 5 mM DTT, 0.1 mM EDTA, 10% (v/v) glycerol) plus ATP (5 mM). Protein concentration, measured by Bio-Rad protein assay in comparison to BSA standards, was typically 12–15 mg/ml. After preparation, cytosol was immediately snap frozen and stored in liquid N_2_.

### Cytosol depletion

RLC and SHC were depleted of p97 using a GST-tagged p97-binding fragment of Ufd1, UT6 (amino acids 215–327), as described [60] and GST was used for the mock depletion. Hsc70, Hsp70, Hdj1, Hdj2, Hsp105, and Apg-2 were immunodepleted using an anti-Hsc70 rat monoclonal (#SPA-815; Enzo Life Sciences), an anti-Hsp70 mouse monoclonal (#ADI-SPA-810; Enzo Life Sciences), an anti-Hdj1 rabbit polyclonal (#SPA-400; Enzo Life Sciences), an anti-Hdj-2 mouse monoclonal (#MS-225; Lab Vision/Thermo), an anti-Hsp105 rabbit polyclonal antibody (#ab24503; Abcam) and an anti-Apg-2 mouse monoclonal antibody (#A-7; Santa Cruz) respectively. For mock depletions, normal mouse, rat or rabbit IgG was employed. Antibodies were covalently coupled to protein G-sepharose (GE) with DMP (Pierce/Thermo) to achieve 2–10 mg antibody per ml resin. 100 µl of antibody-coupled beads were added to 800 µl RLC or SHC, and incubated for 2 h with rotation at 4°C. Beads were then recovered and the supernatant was added to fresh antibody-coupled beads. This process was repeated four times. To determine the extent of depletion, cytosol preparations (20 µg) were fractionated by SDS-PAGE and the concentration of the protein of interest was determined by quantitative immunoblot in comparison to known quantities of pure protein. Typically, >93% depletion of the target protein was achieved. From these studies, the concentration of Hsc70, Hdj1 and Apg-2 in 10 mg/ml RLC was estimated to be: 15 µM, 1.2 µM, and 4.2 µM respectively. Similarly, the concentration of Hsc70, Hdj1 and Apg-2 in 10 mg/ml SHC was estimated to be: 12 µM, 1 µM, and 3.8 µM respectively. Thus, in add-back experiments (Figure 2), pure Hsc70, Hdj1 and Apg-2 were added back to the appropriate depleted cytosol at these concentrations.

### Protein purification

Sse1, Ssa1 and Sis1 variants were generated by Quikchange Lightning Site-Directed Mutagenesis (Agilent). Hsp104 [18], Ssa1 [18], Sis1 [18], Ydj1 [18], Sse1 [49], Apg-2 [49], Fes1 [68], Snl1ΔN [69], Sup35 [19], GFP [57], and α-synuclein [81] were purified as described. Hsp70, Hsc70, Hdj1 and Hdj2 were from Enzo Life Sciences. The activity of Hsp70:Hsp40 pairs and Hsp110 chaperones was confirmed by their ability to suppress the aggregation of denatured luciferase as described [10]. The NEF activity of Sse1, Fes1 and Snl1ΔN preparations was confirmed as described [69]. Firefly luciferase and apyrase were from Sigma and creatine kinase was from Roche. The purity of all proteins was determined by SDS-PAGE and Coomassie staining to be >98%. The Hsp104 concentration refers to the hexamer concentration.

### Luciferase disaggregation and reactivation

Luciferase reactivation was performed as described [10]. Briefly, to assemble aggregates, firefly luciferase (50 µM) in LRB plus 8M urea was incubated at 30°C for 30 min. The sample was then rapidly diluted 100-fold into LRB. Aliquots were snap frozen and stored at −80°C until use. Aggregated luciferase (50 nM) was incubated with the indicated components plus ATP (5 mM) and an ATP regeneration system (1 mM creatine phosphate, 0.25 µM creatine kinase) for 0–6 h at 25°C. Cytosol preparations were added to a final concentration of 10 mg/ml. In disaggregation reactions with pure components, Hsp104, Hsp110, Hsp70, Hsp40 or variants were added to a final concentration of 1 µM. In some reactions, the p97 small-molecule inhibitor, DBeQ (Interbioscreen), or the Hsp70/Hsc70 small molecule inhibitor, VER 155008 (Tocris), was added to final concentration of 100 µM. In other reactions, ATP and the regeneration system were omitted, or replaced with AMP-PNP (5 mM). Apyrase (2 U/ml) was substituted for the ATP regeneration system in other experiments. At the end of the reaction, luciferase activity was assessed with a luciferase assay system (Promega). Recovered luminescence was monitored using a Tecan Safire^2^ or Infinite M1000 plate reader. Luminescence measurements were converted into reactivation yield (% of the maximum recoverable luciferase activity) by comparison to the luminescence of known quantities of soluble, native luciferase.

### GFP disaggregation and reactivation

GFP disaggregation was essentially as described [57]. Briefly, to assemble aggregates, GFP (4.5 µM) in GRB was incubated for 15 min at 85°C. GFP aggregates (0.45 µM) were then incubated with the indicated components plus ATP (5 mM) and an ATP regeneration system (as above for luciferase reactivation) for 0–6 h at 25°C. Disaggregation and refolding of GFP was monitored by fluorescence at 510 nm upon excitation at 395 nm. Fluorescence measurements were converted into reactivation yield (% of the maximum recoverable GFP fluorescence) by comparison to the fluorescence of known quantities of soluble, native GFP.

### Sup35 prion disaggregation

Sup35 prion disaggregation was performed as described [19], [20]. Full-length Sup35 (10 µM) was assembled into prions capable of converting [*psi^-^*] yeast cells to [*PSI^+^*] by incubation in assembly buffer (AB; 40 mM HEPES-KOH pH 7.4, 150 mM KCl, 20 mM MgCl_2_ and 1 mM DTT) plus 10% (v/v) glycerol and GTP (1 mM) for 16 h with rotation (80rpm on a mini-rotator, Glas-Col) at 25°C [19], [20]. Sup35 prions (2 µM Sup35 monomer) were then incubated in AB plus ATP (5 mM) and ATP-regeneration system with the indicated combination of Hsp104 (0.5–2 µM), Sse1 (2 µM or 20 µM), Ssa1 (2 µM or 20 µM) and Sis1 (2 µM or 20 µM) for 6 h at 25°C. Prion disaggregation was then assessed by determining the percentage of the total Sup35 that was soluble in 2% SDS (Sup35 prions resist dissolution by this concentration of SDS) or by the fluorescence of the amyloid-diagnostic dye Thioflavin-T (ThT) (excitation: 450 nm; emission: 482 nm) as described [19], [20], [74].

### α-Syn fiber disaggregation

α-Syn fiber disaggregation was performed as described [17]. Thus, α-syn (80 µM) was assembled into amyloid in AB for 48 h with agitation (1,400rpm in an Eppendorf Thermomixer) at 37°C. α-syn fibers (0.5 µM monomer) were then incubated for 6 h at 37°C in AB plus ATP (5 mM) and ATP-regeneration system without or with the indicated combination of Hsc70 (10 µM), Hdj1 (10 µM), Hsp70 (10 µM) and Apg-2 (10 µM) in the absence or presence of Hsp104 (2.5 µM or 10 µM). Fiber integrity was then determined by sedimentation analysis: reactions were centrifuged at 436,000 g for 10 min at 25°C. Supernatant and pellet fractions were then resolved by SDS-PAGE and processed for immunoblot. The percentage of the total α-syn in the pellet was determined by densitometry and comparison to know quantities of α-syn [17]. Alternatively, α-syn fiber integrity was assessed by ThT fluorescence [17].
